# Unique Digital Images as Incentives in Clinical Trials: A Digital Shift Toward Meaningful Participation

**DOI:** 10.2196/88022

**Published:** 2026-06-08

**Authors:** Xavier Tadeo, Gyula Seres, Peter Wang, Yoann Sapanel, Alexandria Remus, Jasmine L Eyal, Jason Kai-Wei Lee, Simon Chesterman, Julian Savulescu, John Eu-Li Wong, Christopher L Asplund, R Brian Stone, Benjamin C K Tee, Reza Shokri, Marlena Raczkowska, Li Ming Chong, Siong Peng Kwek, Agata Blasiak, Dean Ho

**Affiliations:** 1 Institute for Digital Medicine (WisDM), Yong Loo Lin School of Medicine, National University of Singapore, 28 Medical Drive, #05-COR, Singapore, SG.01, 117456, Singapore, 65 66017766; 2 The N.1 Institute for Health (N.1), National University of Singapore, Singapore, Singapore; 3 Department of Biomedical Engineering, College of Design and Engineering, National University of Singapore, Singapore; 4 Singapore’s Health District @ Queenstown, Yong Loo Lin School of Medicine, National University of Singapore, Singapore; 5 Heat Resilience & Performance Centre, Yong Loo Lin School of Medicine, National University of Singapore, Singapore; 6 Human Potential Translational Research Programme, Yong Loo Lin School of Medicine, National University of Singapore, Singapore; 7 Department of Physiology, Yong Loo Lin School of Medicine, National University of Singapore, Singapore; 8 Faculty of Law, National University of Singapore, Singapore; 9 NUS AI Institute, National University of Singapore, Singapore; 10 Murdoch Children's Research Institute, , , Royal Children's Hospital Flemington RoadParkville, Victoria, Australia; 11 Centre for Biomedical Ethics, Yong Loo Lin School of Medicine, National University of Singapore, Singapore; 12 Oxford Uehiro Centre for Practical Ethics, Faculty of Philosophy, University of Oxford, Oxford, United Kingdom; 13 Department of Medicine, Yong Loo Lin School of Medicine, National University of Singapore, Singapore; 14 Department of Haematology-Oncology, National University Cancer Institute, National University Hospital Singapore, Singapore; 15 Department of Social Sciences, Yale-NUS College, Singapore; 16 Division of Industrial Design, College of Design and Engineering, National University of Singapore, Singapore; 17 Department of Materials Science and Engineering (MSE), National University of Singapore, Singapore; 18 Institute for Health Innovation & Technology (iHealthtech), National University of Singapore, Singapore; 19 Department of Electrical and Computer Engineering (ECE), National University of Singapore, Singapore; 20 Department of Computer Science, School of Computing, National University of Singapore, Singapore; 21 Department of Pharmacology, Yong Loo Lin School of Medicine, National University of Singapore, Singapore; 22 The Bia-Echo Asia Centre for Reproductive Longevity and Equality, Yong Loo Lin School of Medicine, National University of Singapore, Singapore

**Keywords:** digital health, clinical trial engagement, nonfungible tokens, NFT, behavior change, participant incentives, decentralized health care, artificial intelligence, AI

## Abstract

Incentivization in clinical trial participation can be challenging, with many studies failing to meet recruitment or retention goals despite traditional compensation strategies. Digital health evolves, and with it, new approaches can emerge to engage participants meaningfully. We propose unique digital images as a novel, symbolic incentive for clinical trials. Digital images combine qualities such as personalization, ownership, and digital visibility, which may drive engagement more effectively than monetary rewards alone. In our illustrative study, participants complete artificial intelligence–personalized digital therapeutics training using CURATE.DTx, generating individualized learning trajectories. These are transformed into digital artworks and minted as nonfungible tokens given as a reward upon trial completion. This concept integrates gamification, personalization, and blockchain technology to support both intrinsic and extrinsic motivation. We explore the implications for decentralized health care, long-term behavior change, and participant recognition in the context of preventive medicine and longevity science. Our aim is to encourage research into the use of digital incentives to transform the trial participant experience and promote sustained engagement in health interventions.

## Introduction

Persistent challenges in recruitment and retention remain the greatest bottlenecks in conducting clinical trials [1]. Poor recruitment is the main source of discontinuation of trials, representing 9.9% of interruptions [2]. Monetary compensation and access to new therapies are often insufficient to entice individuals to participate in studies. An empirical analysis found that 19% of trials are either terminated for unsuccessful accrual or completed with less than 85% expected enrollment [3]. Extended timelines, increased costs, underpowered analyses, and trial failure are the main consequences. Collected data may be wasted, and public trust may be undermined as participants contribute their time without meaningful outcomes [4]. Treatments are delayed, and funding opportunities might be lost [5]. This raises the issue of whether traditional incentives are sufficient to sustain participant engagement. Communication techniques, while prevalent, do not show robust improvements. A Cochrane review of 68 trials summarized that timely reminders are a useful addition with limited gains, but even customized communication is insufficient to yield improvements over monetary and nonmonetary participation incentives [6]. These limitations suggest that recruitment and retention are fundamentally behavioral challenges, motivating exploration of alternative incentive structures beyond traditional monetary compensation.

We propose a digital age alternative that merges behavioral science and personalization: digital images as personalized trial incentives. A unique, ownable artifact is a durable extrinsic cue that can be internalized into identity (eg, “I am a longevity pioneer”), a psychological process known as self-signaling [7]. Experimental evidence shows that forgoing monetary rewards to earn a symbolic credential boosts pride and sustained prosocial behavior by reinforcing diagnostic self-inference of valued traits [8]. In the context of clinical trials, a singular digital artwork cocreated from personal performance data serves as an identity anchor, transforming transient participation into a lasting narrative of agency and contribution. Moving toward digital and personalized incentives is timely. A nearly universal adoption of electronic health records in hospitals in high-income countries means that new digital incentive models do not require stand-alone platforms. More importantly, the adoption of patient-facing infrastructure mainly through mobile apps is gaining traction [9,10].

Discussion of value, ownership, and participant agency within decentralized health care systems is warranted. To our knowledge, no precedent exists for using artwork as an incentive in clinical trials. This approach leverages the intrinsic value of art to motivate participation and can find application in everyday digital health use. Gamification in the form of digital rewards can improve adherence to health and wellness programs, changing behaviors that are central to healthy aging and longevity.

At its core, this viewpoint piece aims to explore the feasibility and rationale of using personalized digital artwork as a novel form of incentivization in clinical trials. We examine the conceptual foundation, behavioral implications, and potential applications. Our goal is to initiate a broader conversation with digital health researchers, clinicians working at the intersection of health care and technology, developers, and designers of digital health tools on how digital incentives may reshape participant engagement in the era of digital and decentralized health care. We outline the design of a forthcoming feasibility study (Avatar.DTx) as an illustrative example of trialing digital images as participation incentives in clinical research. Accordingly, no empirical results from this trial are reported in this manuscript.

## Conventional Incentives in Clinical Trials

Monetary payment for participating in research has been documented for more than 100 years. In the United States, monetary and nonmonetary compensation to trial participants became mainstream in the 1920s and 1930s, including meals and transportation [11]. Today, most organizations pay for participation in a wide range of human subject research types, but there is still no consensus regarding the adequacy of it. Different commentators on trial compensation define it as wrong, coercive, fair, or necessary [12-14]. Additionally, there is minimal guidance for researchers and institutional review boards (IRBs) to decide whether and how much to pay participants. A long history of research participant compensation has not yielded consensus on the effectiveness, ethics, or design of monetary incentives in clinical trials [15].

Investigators may offer monetary payments to research participants as an incentive to take part, as fair compensation for their contribution, and/or to offset related financial burden. However, there is limited evidence on the necessity of monetary incentives to boost recruitment. Healthy individuals are attracted by money but also for other reasons, such as altruism, curiosity, and attention from physicians [16-18]. Patients affected by the researched condition are often interested in the interventional treatment that could benefit their health, although little research has been conducted on the influence of money in their decision to participate [19]. Money does not ensure more recruitment diversity either given the distrust of the research establishment by some ethnic groups [20]. The American Academy of Pediatrics recommended giving gifts to participating children instead of money following an appreciation model [21]. As we can see, the form and amount of payment can be adjusted depending on the type of research and participants, their contributions and vulnerabilities, local guidelines, and cultural norms. This choice matters: it shapes both recruitment volume and demographic composition. In US studies, the share offering any compensation varies widely (approximately 35%‐63% across trial phases and 10%‐84% across intervention types), underscoring heterogeneity by design and risk profile [22].

According to Bentley and Thacker [23], monetary payments appear to achieve their intended effect of increasing participants’ willingness to enroll in research. However, payments could compromise the integrity of study results. First, monetary compensation is associated with increased motivation to participate in research independently of perceived risk, with higher payments further amplifying motivation. Second, higher monetary payments may create incentives for participants to withhold or conceal information regarding restricted activities. The authors conclude that further research is needed to inform sound policies on compensating research participants [23]. Similarly, Treweek et al [6] evaluated strategies to improve recruitment to and retention in randomized controlled trials—studies in which participants are randomly assigned to an intervention or control group to assess causal effects. They concluded that the evidence base remains limited, heterogeneous, and highly context dependent. Recruitment and retention challenges are multifactorial behavioral phenomena influenced by perceived burden, trust, logistical barriers, and expectations of benefit rather than purely by financial considerations. Monetary payments may improve follow-up rates, particularly for low-burden tasks such as survey completion; however, their impact on initial enrollment is inconsistent, and optimal structures, amounts, and delivery mechanisms remain poorly defined. The reviews also highlight the scarcity of rigorous evaluations of innovative engagement strategies, noting that most tested interventions predate contemporary digital and decentralized trial models [4].

Fixed payments may inadequately reflect the value and opportunity cost of participation that varies widely between individuals. Evidence from behavioral economics shows diminishing marginal returns beyond modest thresholds [24]. Monetary incentives often trigger an undesirable “crowding-out” effect where extrinsic rewards dilute intrinsic motivation, especially in altruistically framed activities such as research participation [25,26]. They rarely leave a lasting impression on the participant.

Clinical research increasingly shifts toward remote participation, digital therapeutics—software-driven, evidence-based interventions designed to prevent, manage, or treat medical conditions—and app-based data collection. Consequently, traditional retention tools may be insufficient to sustain long-term engagement. This gap underscores the need for novel, context-appropriate incentive mechanisms specifically designed for modern digital health trials.

## Digital Art Incentives

Digital art paradigms such as scarcity, provenance, authorship, and symbolic value shape the meaning attributed to cultural objects, often independently of their direct monetary value [27]. In the context of clinical trials, digital art can function as an incentive, offering recognition, identity, and engagement. Moreover, art has always been relational in varying degrees, a factor of sociability and dialogue, adding another nontransactional layer to this incentive type [28]. Digital art has entered mainstream culture, with the art ecosystem recognizing its value. Among global art forms, digital art saw the biggest uplift in participation and spending in 2024 to 2025. Art collectors are growing comfortable with hybrid forms of exchange [29].

From the perspective of digital health trials, digital art as an incentive presents the advantage of high compatibility with digital infrastructures. High internet penetration and health care data digitalization can enable digital art incentives to be delivered, tracked, and experienced with minimal friction.

Tokenization technologies such as nonfungible tokens (NFTs) represent a convenient implementation platform for digital art–based incentives. Blockchains use fungible tokens that are not only currency but also utility mechanisms that align incentives among participants. Blockchain computation has an economic cost, and the token is the “fuel” that enables operations, tying economic value to network use. Users pay in the native token to execute code, and validators are compensated in the same token for processing transactions. Tokens are incentives for all stakeholders and allow the decentralized network to be sustained [30,31]. NFTs share this principle but, by virtue of being noninterchangeable (unique), can encode properties such as participation, ownership, or rights. NFTs are tools for structuring participation and verifying contributions in distributed systems [32,33].

In a digital health trial context, where incentive alignment is critical, NFTs enable incentive design beyond conventional monetary compensation. They are programmable and can encode participation, provenance, and conditional rights within a clinical research protocol. Blockchain digitally certifies ownership of unique digital assets, and NFT ownership paths are public. This allows behavioral patterns to be inferred (eg, a trial participant’s relationship with their tokenized incentive, including engagement, perceived value, and longitudinal interaction).

Since their inception, NFT market activity has concentrated on a few assets and collections, which indicates that engagement and loyalty are bound to certain art ecosystems [34]. In the context of digital health trial incentives, this pattern reveals the significance of aligning incentives with participants’ identities and preferences: sustained engagement is more likely to appear from personalized and meaningful incentives.

Monetary rewards may crowd out intrinsic motivation by framing participation as a transactional exchange: payment in exchange for participation. In contrast, a digital artwork, when derived from an individual’s own performance or contribution, is expected to carry an intrinsic valuation. This is strengthened by its signaling value. The primary mechanism lies in the act of earning the artifact through task completion: its uniqueness and direct linkage to study participation confer meaning tied to achievement. This characteristic is lacking in other discussed conventional forms of participation incentives. The design allows participants to derive value from their actions (“I completed this training; therefore I am committed”), consistent with self-signaling theory [7]. Public visibility, such as the ability to share the artwork, may reinforce the identity signal, although it is not required for the mechanism to operate [8].

From our perspective, incentive art used in a clinical trial belongs to a distinct category from speculative digital art. Its economic and behavioral properties are shaped by purpose and context rather than pecuniary motives. Tokenized or not, digital incentives are different from cash payments, with advantages and risks worth considering in trial incentive design.

## Conventional, Digital, and Tokenized Incentive Mechanisms

Traditional, nontokenized, and tokenized incentives differ in technical implementation and behavioral properties (Table 1). Monetary payments are immediate, bounded in value, and aimed to offset the inconveniences of participation [35]. Their impact on recruitment and retention remains uncertain, and they carry a potential risk of coercion. Nontokenized digital incentives (eg, digital art, certificates, and badges) are nonmonetary and nontransferable and find value through recognition, identity signaling, and engagement [36]. Tokenized digital incentives are unique, programmable, and able to encode contribution, identity, and provenance. Their value is derived mainly from symbolic meaning and personalization [37].

**Table 1.** Comparison of conventional, digital, and tokenized incentive mechanisms in clinical trials.

Feature	Conventional cash incentives	Nontokenized digital incentives	Tokenized digital incentives
Fungible	Yes	Yes	No
Secondary market	No	No	Yes
Identity signaling	No	Possible	Possible
Long-term value	No	Contextual	Potential
Primary incentive logic	Compensation	Recognition	Recognition
Ethical risk	Coercion	Exclusion	Financialization

Nontokenized incentives leverage digital infrastructures without introducing market dynamics. They may have long-term value only in a personal, symbolic context. They introduce an ethical risk among participants with differing levels of digital literacy or cultural expectations, who may perceive symbolic or digital rewards as insufficient recognition of their contribution [38]. Tokenized incentives enable traceability and attribution with a secondary market. This feature introduces the risk of financialization, a shift in participant motivation from engagement to profits. However, incentive design (nontransferability and personalization) can mitigate such behavior.

The motivational spectrum that conventional, nontokenized, and tokenized digital incentives occupy is different. Money is immediate and simple but not meaningful. Nontokenized digital incentives emphasize symbolic value, recognition, and contextual meaning without direct economic relevance. Tokenized digital incentives have behavioral, social, and symbolic components, and they may require governance to avoid market-driven distortions. Due to their different incentive mechanisms, they can be complementary rather than substitutionary. Their effectiveness and ethical profile depend on their design and contextual deployment.

## Digital Images as Incentives: Methodological Recommendations

Trials should include both quantitative and qualitative measures to assess the feasibility and impact of using digital art as a participation incentive in clinical research. Suggested outcomes include completion rates, participant retention, artwork retention or trading behavior, and qualitative feedback on acceptability, perceived value, and motivational impact collected through structured interviews or surveys. It is critical to document participants’ prior familiarity with digital assets to help account for selection effects and interpret responses. When implementing incentives as NFTs, trials should also report technical implementation details, including wallet setup, data deidentification procedures, and blockchain transaction protocols. Potential biases such as digital literacy disparities, speculative motivations, and unequal access must be anticipated and addressed in the design. In addition, studies should document governance and life cycle considerations related to digital asset ownership, such as procedures for handling loss of access, transferability constraints, or ownership disputes as these factors may influence participant experience and long-term acceptability.

To reduce the risk of speculative participation, all materials—especially consent forms—should clearly state that the artwork is intended as a nonfinancial participation incentive with no guaranteed market value. This helps manage expectations and preserve intrinsic motivation. Unequal access can be minimized by avoiding the requirement of prior familiarity with digital art and/or blockchain technologies and using accessible platforms with minimal transaction costs. Clear communication about privacy safeguards, technical support availability, and poststudy access to the digital artwork is also important to ensure equitable participation and sustained trust. Together, these strategies ensure that participation reflects interest in the intervention rather than in external incentives alone.

## An Illustrative Example: The Avatar.DTx Study

Our team has conceived a methodological study to assess the feasibility and acceptability of using images as rewards in a digital therapeutics trial for cognitive training. This pilot trial was approved by the National University of Singapore IRB (NUS-IRB-2023-183) and has been registered on ClinicalTrials.gov (NCT06647030). Recruitment has not started. This planned trial is an example of how our proposal can be tested (Multimedia Appendix 1).

Trial participants will use CURATE.DTx, a tablet-ready, multitasking serious game successfully investigated in previous clinical trials (NCT04848935 and NCT06240897) [39,40]. CURATE.DTx leverages a small-data (ie, using individual longitudinal data rather than population-scale data), AI-derived platform (CURATE.AI) to dynamically personalize cognitive training by adjusting game intensity. Performance data from each participant are used to generate a unique learning trajectory profile. Once the profile is generated, it allows for real-time adaptation of training inputs to optimize individual learning outcomes. As a result, CURATE.DTx offers a promising method for enhancing cognitive training and potentially preventing age-related cognitive decline. Its interface is a modified version of the Multi-Attribute Task Battery (MATB), the Online MATB [41-43]. This MATB version is an update of the MATB flight deck simulator originally developed by the National Aeronautics and Space Administration and the US Air Force, which was previously leveraged by our research group [44-47].

**Figure 1.**
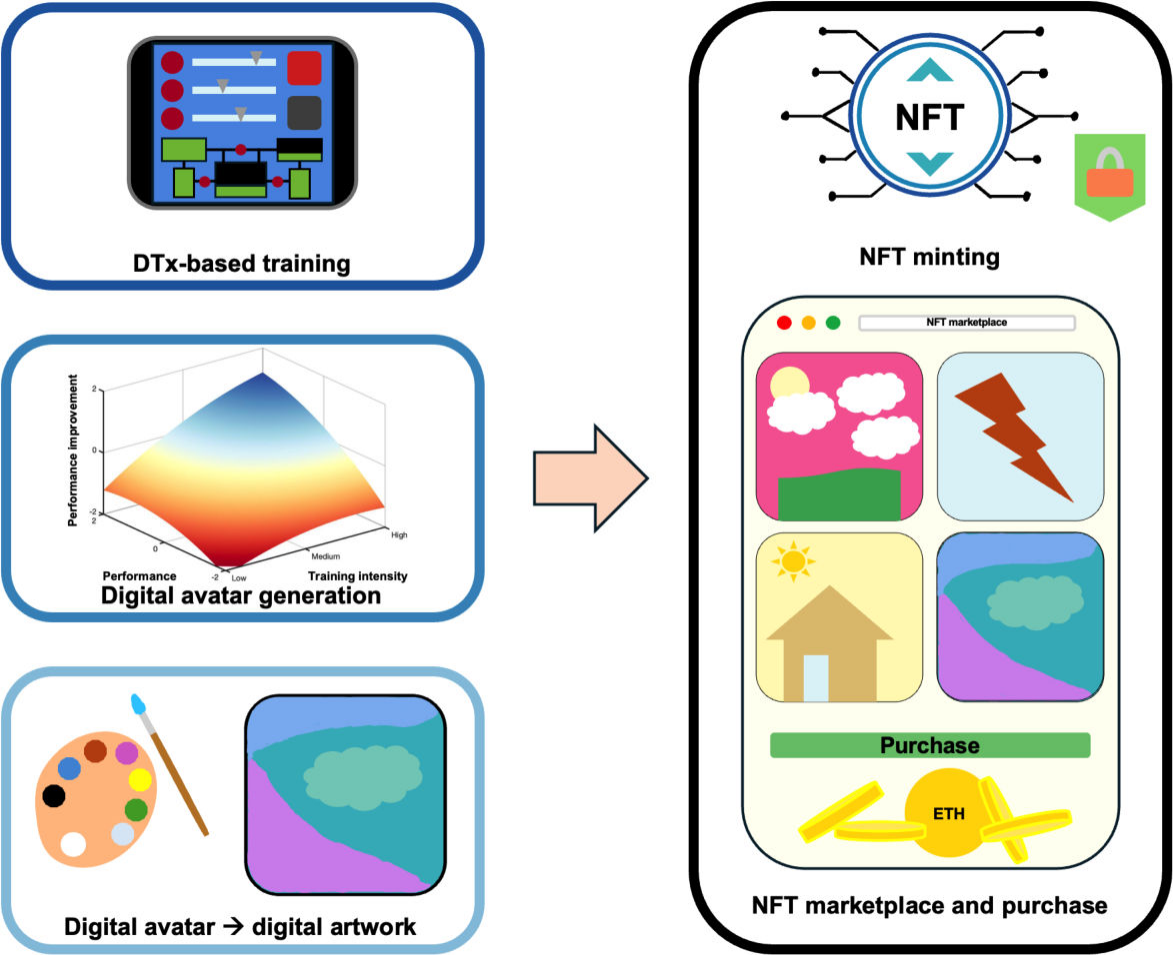
Overview of NFT minting and transfer in the planned Avatar.DTx study. Participants complete CURATE.DTx training, after which deidentified performance data are used to generate personalized digital artwork that is minted as an NFT and transferred to the participant’s digital wallet. ETH: Ether; NFT: nonfungible token; DTx: digital therapeutics.

Figure 1 shows an overview of our study. A total of 15 healthy participants will engage with CURATE.DTx as part of a structured cognitive training program. The platform will capture personalized performance data across a 10-week training period. Those data will then be transformed into a unique, generative artwork by a digital artist. Upon completion of the study, the artwork will be available as a high-resolution image file and, additionally, minted as an NFT and transferred to the participant’s digital wallet. The images in this study will be integrated into a gamified, meaningful process. If participants do not complete the full training regimen, they will be compensated modestly per session in local currency, but they will not receive anything else. This structure is expected to reinforce engagement without coercion.

A relevant feature of these images is that they will be cocreated by a human artist and a generative artificial intelligence (AI) model. Although technically they are both unique digital art formats, human-made art is perceived as more valuable [36,48]. This way, participants also receive an otherwise paid service for free, further driving up the perceived value of an artwork.

**Figure 2.**
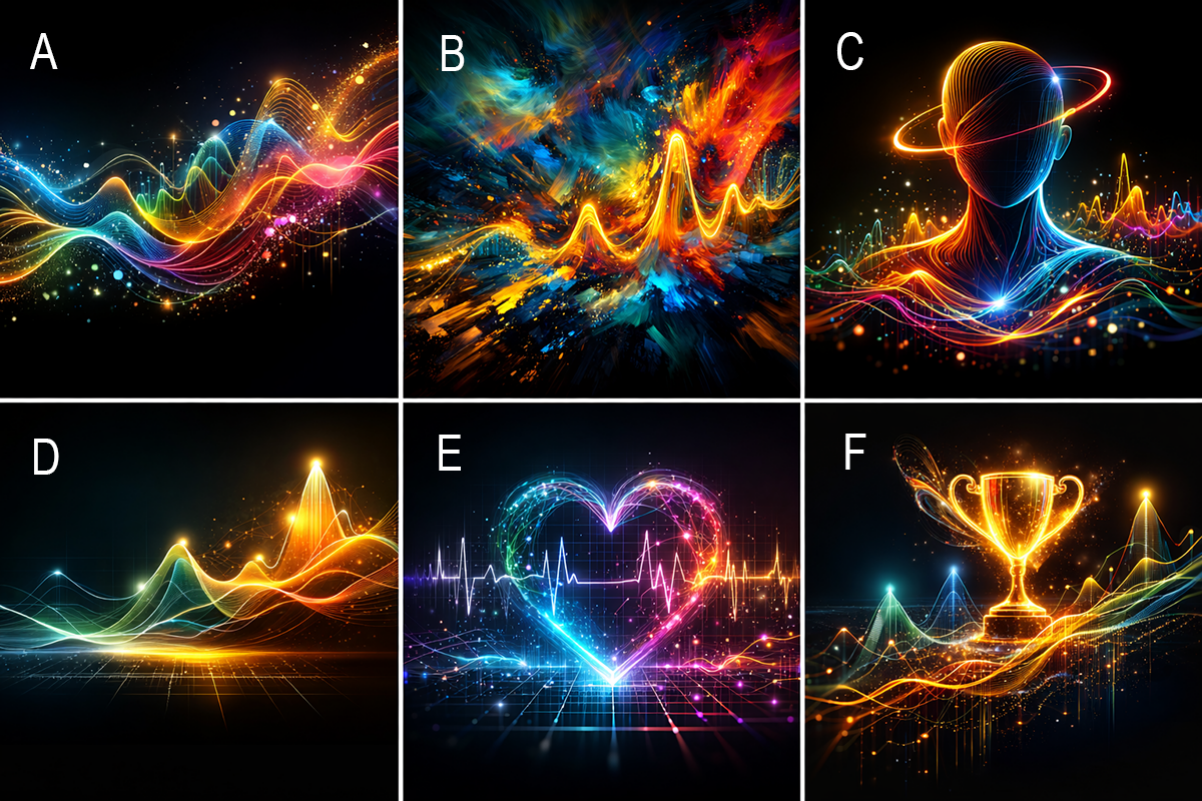
Stylistic variants of personalized digital artworks illustrating outputs that could be generated under a human-guided artificial intelligence (AI) applied to abstracted performance features, including (A) abstract, (B) painterly, (C) generative lines (avatarlike), (D) minimal, (E) biometric inspired, and (F) symbolic milestone representations. In the planned Avatar.DTx trial, a human digital artist will intervene in the artwork creation process by guiding the AI (selecting inputs and adjusting aesthetic parameters); the images shown in this figure are fully AI generated and included solely for illustrative purposes. All images are derived from synthetic data and do not contain real participant information or clinical measurements.

The digital artist will use models such as generative adversarial networks and variational autoencoders. The algorithm will introduce noise to ensure variability and prevent identical outputs, and the artist will shape the creation by selecting inputs, adjusting parameters, and polishing results. Noise will operate as a source of controlled randomness, enabling each run to yield distinct variations. For example, when transforming a user’s profile into a digital painting, the artist may guide choices of color schemes, background motifs, or line styles while the algorithm determines their precise configuration (Figure 2).

Our main goal is to assess the feasibility and acceptability of digital incentives as a means of encouraging participation. We will conduct in-depth interviews to understand perceptions of human art through digital means, AI-generated art, NFTs, ownership, value, and privacy. Technical and ethical safeguards will be put in place, including data anonymization and transparent consent.

## Broader Implications

The potential effects of using digital image–based incentives go beyond recruitment and retention in clinical research, with implications for mechanisms that sustain adherence to health care interventions. At present, the role of AI and data sciences in the emerging fields of healthy aging and human longevity is being widely explored [49-52]. Longevity science and preventive health interventions increasingly rely on behavior change over long periods [53]. Maintaining long-term engagement in physical training, dietary interventions, and cognitive training activities is a major challenge [37]. Devising lasting commitment strategies becomes essential. Digital images, especially when linked to personalized feedback (eg, AI and small-data analytics generation of individualized health profiles), offer a new class of motivational tools for decentralized and digital health. Additionally, the integration of data-driven self-optimization with digital art incentives can foster a digital community where users share milestones and motivate each other [54].

Beyond their incentive value, the practical relevance of digital images in clinical contexts depends on whether they can be implemented within existing health care infrastructures without adding operational or technical burden. AI-based systems are increasingly used to generate or analyze clinically relevant visual outputs across medical domains, including applications in dental imaging and aesthetic planning [55,56]. Digital images can be incorporated into existing clinical systems without changing the core electronic health record. Rather than embedding new software directly, a separate incentive support tool can connect to it using widely adopted health data standards (eg, Fast Healthcare Interoperability Resources) [57,58]. In practical terms, this tool would mitigate privacy concerns by accessing only the limited information necessary to inform incentive selection. For example, appointment history leads to the generation of a recommendation about the type of incentive, when it should be offered, and how it should be communicated (eg, via patient portal message or SMS text messaging). Recommendations can be delivered within the clinician’s existing workflow so that they appear as part of routine care processes, such as after a missed appointment or during discharge planning. Digital images as incentives or tokens of appreciation can be seamlessly integrated in these systems.

This approach allows incentive personalization to operate as an extension of current systems. Safeguards must include maintaining records of recommendations, allowing clinicians to override automated suggestions when appropriate, limiting data use to what is strictly necessary, and monitoring for unintended disparities in how incentives are distributed across patient groups [59].

Digital artwork may also hold promise for underrepresented populations seeking greater personalization or for individuals drawn to new forms of digital participation [60].

It is worth noting that digital incentives are not necessarily exclusive; they can complement rather than replace traditional approaches to participant motivation such as financial compensation or altruism. Artistic forms of compensation add aesthetic and symbolic value. A culture of participant-centered trial conduct is emerging. Within that progression, rewarding participation with personalized digital art represents a coherent step.

Evidence shows that the value of artworks is driven by 2 factors: the intrinsic enjoyment dividend and additional trade value [61]. While we intend to present digital images as primarily nonspeculative incentives, enabling the trade of images in the form of NFTs may increase perceived value for some participants without being a necessary or primary feature of the incentive design. This can be facilitated by platforms such as OpenSea or Rarible, which make the process accessible to less technology-savvy participants.

## Ethical and Practical Considerations

Using digital artworks as incentives in clinical research introduces new ethical and methodological questions. When using NFTs, ethical concerns about data visibility, even in abstract artistic forms, require serious consideration. Privacy-preserving algorithms, transparent consent procedures, and fallback contingencies should be implemented. During the consent process, participants should be informed that, if they do not complete the full training regimen, they will receive modest per-session compensation in local currency without eligibility for further incentives. This approach is designed to support engagement while avoiding undue inducement. No personal or clinical data should be stored on-chain. Only minimal metadata—such as a token ID and a link to the digital artwork hosted off-chain—should be recorded on the blockchain [62]. All sensitive participant data should be stored securely off-chain on encrypted institutional servers in accordance with local data governance policies.

To minimize the cognitive burden associated with blockchain interactions, researchers should manage e-wallets on behalf of participants and facilitate onboarding into digital art platforms. To account for the immutability of blockchain records, NFTs should only be minted and transferred after participants complete the study or intervention. If a participant withdraws prior to study completion, no token should be created or assigned. While there are many blockchains available, Ethereum is a sensible choice for NFT minting due to its mature infrastructure and wide support for ERC-721 standards [52]. Since Ethereum’s transition to a proof-of-stake consensus mechanism in September 2022, validators secure the network by staking 32 ETH, eliminating the need for energy-intensive mining [63]. This upgrade has reduced Ethereum’s energy use by over 99%, improving sustainability [64] and demonstrating that it is possible to choose a blockchain with relatively low environmental impact.

Intrinsic value is inherently random and not directly observable. It varies from person to person depending on individual taste, familiarity with digital art, and perceived emotional or aesthetic resonance [65]. Because of this subjectivity, predicting or standardizing the perceived worth of a digital artwork is difficult. Nonetheless, if the potential value of the incentive is disclosed in advance, it may introduce a selection effect among participants, attracting individuals motivated primarily by financial interest [66]. Such selection effects could bias recruitment and compromise the generalizability of findings. Furthermore, the perceived transactional nature of the incentive could risk undermining intrinsic motivation for behavior change [67]. To prevent a speculative motivation bias whereby participation incentives are dominated by a perception that an NFT is a tradable asset with a certain upside potential, the study protocol must clearly address this by avoiding investment framing, avoiding recruitment channels associated with trading, and controlling for the participants’ motivation to join in an exit questionnaire.

The prospect of receiving a tradable asset, even if symbolic, may also exert undue influence among participants unfamiliar with the underlying technology [32,68]. This intersects with concerns of equity as individuals with lower digital literacy may face barriers to understanding, accessing, or benefiting from NFT-based incentives. Additionally, NFTs exist within markets characterized by speculative value and volatility, potentially leading to misconceptions about future worth or resale opportunities [69]. These risks are not inherent to digital incentives themselves and can be mitigated through incentive design and governance, including nontransferability, delayed minting until study completion, and IRB oversight to ensure proportionality and transparency.

Questions regarding data governance, intellectual property (IP), and platform dependence remain unresolved, especially in the context of health care research [70]. Careful data minimization; explicit IP disclosure; and the use of open, portable technical standards can substantially mitigate these risks.

Due to the absence of centralized regulation and standardized licensing practices, a commonly cited issue with NFTs is that buyers often obtain only a token reference (eg, a link to a file) rather than rights to the underlying IP, in this case, the artwork, potentially eroding public trust [71]. In clinical trials, IRB oversight may help mitigate these concerns by ensuring clear and ethical disclosure of compensation and related IP rights. In this setup, the artwork serves to incentivize participants in clinical trials, and IRB oversight ensures proper disclosure of any IP-related compensation.

Recruitment has not started.

## Conclusions

We encourage digital health and clinical research communities to explore incentive models that are more than transactional. Digital art and other forms of meaningful recognition can reformulate participant incentives for contributing to science. In clinical trials, where recruitment, retention, and sustained participant engagement directly determine scientific validity, incentive design remains a persistent and unresolved challenge.

This exploration is particularly timely. Longevity science and preventive health interventions hinge on adhering to diet; exercise; cognitive training; and, when necessary, medication. However, maintaining engagement over time is still a challenge. Digital incentives that combine personalization, perceived value, and public visibility provide a new tool to support both clinical research and long-term interventions.

Health care is shifting toward proactive and personalized models, and the mechanisms for motivating and rewarding individuals must evolve alongside it. Beyond the immediate benefits of recruitment or adherence, digital incentives raise broader questions about recognition and reciprocity in research. In the future, individuals may expect forms of engagement that acknowledge their role as active contributors rather than passive participants. When trial participants receive a unique artifact of their involvement, they are acknowledged. Health care is becoming more digital, allowing for new forms of engagement and reward. Digital images may be one such form.

## Supplementary material

10.2196/88022 Multimedia Appendix 1 Avatar.DTx methodological details.

## Abbreviations

AI: artificial intelligence
IP: intellectual property
IRB: institutional review board
MATB: Multi-Attribute Task Battery
NFT: nonfungible token
