# Supplementary material for: Unique Digital Images as Incentives in Clinical Trials: A Digital Shift Toward Meaningful Participation
Source: J Med Internet Res. 2026 Jun 8;28:e88022. doi: 10.2196/88022 (PMC13245546; doi:10.2196/88022)
Supplement: Multimedia Appendix 1 [file jmir-v28-e88022-s001.docx]

**Avatar.DTx Methodological Details**

**CURATE.DTx Platform**

CURATE.DTx is a digital therapeutics platform that provides personalized cognitive training through a tablet-based adaptation of the Multi-Attribute Task Battery (MATB), a flight deck simulator originally developed by the National Aeronautics and Space Administration (NASA) and subsequently adapted by the United States Air Force (USAF). The MATB platform is a multitasking system in which participants must simultaneously manage four tasks: Systems Monitoring, Communications, Tracking, and Resource Management. In Systems Monitoring, participants must react quickly when indicators illuminate; response accuracy and speed are the dependent measures. Communications involves listening to audio commands and responding only to the call sign “NGT504” by adjusting the radio station and frequency as instructed, while ignoring other call signs and commands. The dependent measures of these responses are speed and accuracy. In Tracking, participants have to keep a moving ball as close to the center of a crosshair as possible by clicking within the field to correct deviations. In Resource Management, participants must keep the fuel levels of two tanks within a specific range by opening and closing valves and compensating for valves that intermittently close or fail. For Resource Management and Tracking, the dependent measure is

root mean square deviation from the target fuel level or the target position relative to the crosshair. Each task is controlled by a script of event timings and difficulty settings

(e.g., tracking difficulty).

CURATE.DTx leverages CURATE.AI, an AI-derived platform that allows to optimize a treatment dose or intensity with a set of individual dose:response data. To personalize MATB cognitive training, CURATE.AI correlates a user’s training difficulty levels (inputs) with that person’s performance outcomes (outputs). A profile is calibrated with this small data set, which is then used to identify the next cycle’s optimal, specific training intensity for that person. Specifically, to calibrate a profile, a participant has to complete a series of testing and training blocks. The first block and every other block (testing blocks) are set at constant medium task intensity to collect performance values and calculate z-scores (difference in performance between testing blocks administered before and after a training block). The training blocks intercalated between testing blocks alternate among low, high and medium task intensity. The CURATE.AI profile represents the performance improvement associated with each training block (z-scores).

**Recruitment**

We will conduct recruitment for this study through word of mouth, online postings, and standard recruitment flyers. Interested participants will be able to contact the research team, and potential participants will be contacted via email or telephone to provide more information about the study. Eligible participants are healthy adults aged 21 and above, with mental capacity determined with the Mini-Mental State Examination (MMSE), and who consent to having their interview audio-recorded. Exclusion criteria include difficulty understanding and reading English —as the digital therapeutics (DTx) text and audio are in English—, significant hearing impairment, evidence or diagnosis of severe cognitive impairment, and an inability to complete the study as determined by the investigators. Our research team will proceed to screen for eligibility at initial contact. Suitable participants will be required to attend an online informed consent session and provide explicit e-consent before commencing the DTx intervention. During this session, an Institute for Digital Medicine (WisDM) research team member, in the presence of a witness, will explain the background and aims of the study and and provide details of trial procedures, risks involved and potential benefits of participating. Participants will be informed during the consent process that completion of the full digital therapeutic program is required to receive the NFT artwork. Participants will be informed that, if they choose to share the NFT on social media or other platforms, they should avoid including personal health details or information that could compromise their privacy. Participants will be allowed to ask as many questions as needed. Participants who agree to take part in the study will digitally sign the Informed Consent Form.

**Data Collection and Analysis**

Each of the fifteen individuals participating in this study will engage in a ten weeks period comprising DTx sessions aimed at creating a digital avatar/NFT, along with a semi-structured interview. Regarding the DTx sessions, participants will interact with the DTx twelve minutes per day, three days per week for ten weeks. For each participant, we will derive a personalized profile, consisting of two-dimensional representations of the relationship between training intensity and performance (see Main Text, Figure 2). A selected digital artist will receive the profiles without any personal or medical data and will create fifteen unique artworks. The artist will then send the artworks to the team. After the last DTx session (regardless of study completion by the participant), a 60-minute semi-structured interview session will be conducted by a team member, either at WisDM in person or via Zoom. All responses will be audio-recorded and transcribed verbatim. We will use thematic analysis of de-identified transcripts to identify emerging or recurring themes. Data will be analyzed using NVivo. The analysis will begin with open/primary coding, where we will descriptively label data. Subsequently, we will group the labels into categories based on literature (i.e., secondary coding). These categories will then help create broader themes/assertions.

**Incentive Generation and Distribution**

The researcher will display the personalized artwork to each participant; however, only those who have completed all the DTx training sessions will receive it. Partial completion will be compensated at a rate of SGD 2.5 per DTx session. This will be communicated as a design feature—not a penalty—and participants will be assured that compensation is not contingent on performance or outcome. Participants will also be advised that the artwork is non-identifiable and derived from anonymized performance data.

The team will mint the NFTs on Ethereum and send them to the participants’ digital wallets (if the participants don’t have a digital wallet, the research team will help them to set one up). The NFTs will be displayed on OpenSea. If the Ethereum blockchain stops its activity within two years of the NFT minting, the team will mint a new NFT for each participant in an alternative blockchain and compensate them with SGD 150.

**Data Storage and Confidentiality**

We will store collected data electronically on an encrypted thumb drive and/or device(s) and a secure cloud database. We will also keep a copy of the experimental data without personal identifiers in a password-protected digital storage facility. Only the P.I. and collaborators will have access to the data. Our team will only collect personal data (name, email address, and contact number) to recruit participants and obtain their consent. We will collect personal data separately from research data and erase it upon completion of the research. Research data will be retained for at least ten years, in line with National University of Singapore Research Data Management Policy DPRT-2011-04, after which we will delete it. Quotes may be published without identifying research participants.

**Blockchain-Specific Considerations and Privacy Risks**

We will mint NFTs in the Ethereum blockchain. As such, an artistic representation based on healthy participant DTx data will be made publicly available, albeit in a non-actionable and non-numerical format. There is a possibility that the shape of the digital avatar may serve as an identifier. This is likely addressed by the fact that the avatar is an artistic rendering that does not contain direct identifiers or reconstructable biometric features.

**Interview Guide**

Demographic Details

- Age
- Gender
- Education
- Occupation/past occupation
- Medical condition if any
- Family status (e.g. who are they living with? Children?)
- Technology use (e.g. mobile phone, television, cryptocurrency, etc.)

Theme 1: Project objective

- Could you tell us why you decided to participate in the study?
- To what extent did the NFT as a remuneration/ incentive for the study motivate you to participate?
- What were your expectations?
- What do you feel about the project (testing the use of NFTs as trial incentives)? Can

you describe what you know about NFTs? And how?

- What were you looking for when you were informed of such a project?
- (If they reject it) Why you didn’t want the NFT?
- (If they didn’t qualify) Why the NFT wasn’t incentive enough to try and complete all

the required sessions?

Theme 2: Overall experience

- Can you describe your experience participating in the study?
- What do you like about the DTx experience and NFT/Digital Avatar creation

experience?

- What don’t you like about the DTx experience and NFT/Digital Avatar creation

experience?

- Did you find any part of the study difficult? Why?
- What was easy through your participation in the study? Why?
- Is there something you’ve done previously that’s similar?
- Would you know anyone who might enjoy this experience? Could you describe those

people?

- Do you like the NFT artwork? Is there any other NFT art form (short film, video loop,

generative art, pixel art, photography, portrait, etc.) you would rather receive?

- Is there any other NFT type (gaming item, metaverse asset, fashion, sports

memorabilia, music, event ticket) you would rather receive?

- Is there any other art medium (physical painting, sculpture, collectible, etc.) you

would rather receive?

- How compensated do you feel with the NFT as compared to receiving cash (e.g.

SGD2.5 per DTx session)?

- Would you be more likely to appear for a long-term assessment (eg. 6-month, 1 year)

of our DTx training outcomes if provided with a second NFT based on all of the

participants’ NFTs?

- Is this something you would like to receive in a clinical trial (not a trial testing the use

of NFTs in trials)?

- Is there something that might stop you from continuing to be part of the study? Why?
- Do you think there were or there could be any adverse events in this practice?
- Is there a way we can improve your experience? Did you feel like you needed help

any point throughout your participation?

- Did the study meet your expectations/initial thoughts about it?

Theme 3: Prospects

- What will you do with the NFT: keep it, display it, trade it?
- Do you think your NFT will go up in value/popularity? And this NFT collection?
- Do you think this will be a common form of trial compensation in the future?
- Do you have any opinions regarding the storage of health/healthcare-related data on the blockchain?
- In a hypothetical situation (This will not be done as part of this study), would you

consider providing consent to having your de-identified data made available on the

blockchain for population-wide health analysis provided you are reasonably

compensated?

- Do you think that blockchain-driven health data sharing should be expanded/further

explored in the event that increased data sharing could reduce healthcare expenditures at a population-scale?
